# Older adult drug overdose: an application of latent class analysis to identify prevention opportunities

**DOI:** 10.1186/s12954-024-00973-4

**Published:** 2024-03-13

**Authors:** Maryann Mason, Kaveet Pandya, Alexander Lundberg

**Affiliations:** 1 Buehler Center for Health Policy and Economics, 420 E. Superior St. 9th floor, Chicago, IL 60611 USA; 2 Department of Emergency Medicine, Feinberg School of Medicine, Northwestern University, 420 E. Superior St. 9th floor, Chicago, IL 60611 USA

**Keywords:** Drug overdose, Epidemiology, Prevention, Substance use

## Abstract

**Background:**

Older adult overdose death rates have increased significantly in recent years. However, research for prevention of drug overdose death specific to older adults is limited. Our objective is to identify profiles based on missed intervention points (touchpoints) to inform prevention of future older adult unintentional overdose deaths.

**Methods:**

We used latent class analysis methods to identify profiles of decedents aged 55 + years in the Illinois Statewide Unintentional Drug Overdose Reporting System. This system collects data on 92.6% of all unintentional overdose deaths in Illinois and includes data from death certificates, coroner/medical examiner, toxicology, and autopsy reports. Data include decedent demographics, circumstances leading up to and surrounding the fatal overdose and details regarding the overdose. Variables in the latent class analysis model included sex, race, alcohol test result, social isolation, recent emergency department (ED) visit, chronic pain, and pain treatment.

**Results:**

We identified three distinct decent profiles. Class 1 (13% of decedents) included female decedents who were in pain treatment, had physical health problems, and had greater likelihood of a recent ED visit before their death. Class 2 (35% of decedents) decedents were most likely to be socially connected (live with others, employed, had social or family relationships) but less likely to have recent healthcare visits. Class 3 (52% of decedents) decedents had higher social isolation (lived alone, unemployed, unpartnered), were mostly male, had fewer known physical health conditions, and more alcohol positivity at time of death. White decedents are clustered in class 1 while Black decedents are predominant in classes 2 and 3.

**Conclusions:**

These profiles link to potential touchpoint opportunities for substance use disorder screening harm reduction and treatment. Class 1 members were most likely to be reachable in healthcare settings. However, most decedents were members of Classes 2 and 3 with less engagement in the healthcare system, suggesting a need for screening and intervention in different contexts. For Class 2, intervention touchpoints might include education and screening in work or social settings such as senior centers given the higher degree of social connectivity. For Class 3, the most isolated group, touchpoints may occur in the context of harm reduction outreach and social service delivery.

**Supplementary Information:**

The online version contains supplementary material available at 10.1186/s12954-024-00973-4.

## Introduction

The drug overdose epidemic, which has plagued the United States for more than the past two decades, continues to evolve. Older adults are increasingly involved in drug overdose deaths [1]. Among adults aged 55 and older, the annual rate of unintentional drug overdose death increased from 1.8 per 100,000 population in 1999 to 23.17 per 100,000 population in 2021 [2]. From 2008 to 2018, the proportion of older adults who entered substance use disorder treatment for the first time increased compared to that of younger adults, and among these first time admissions, admissions for opioid use disorder (OUD) with heroin increased substantially [3, 4]. Emergency department (ED) visits by older adults for opioid use increased by 16% from 2021 to 2022 [5, 6]. From 2005 to 2019, California saw a 1,808% increase in ED visits for cannabis-related issues among those 65 years and older [7]. As the American population ages, the population of older adults at risk for drug-related harms and fatal drug overdose continues to grow [8]. 

Risks for older adults have mirrored the wider the drug epidemic with growing burdens from synthetic opioids and stimulant use [9]. Data from the CDC indicate a 53% increase in overdose deaths involving synthetic opioids other than methadone between 2019 and 2020 in adults over 65 years of age [10]. Though less frequent than opioid-involved deaths, drug overdose deaths involving stimulants (e.g., cocaine and psychostimulants with abuse potential) among older adults are also increasing [2]. The majority of these stimulant-involved deaths occur without opioid-involvement [2]. Furthermore, unlike opioids, there exist no currently proven overdose reversal agents or recovery support medications for these stimulants [11, 12]. 

Despite the availability of naloxone (generic Narcan), an overdose reversal agent, older adults may face access barriers increasing risk for overdose. For example, naloxone was recently approved by the Food and Drug Administration for over-the-counter access. While generally heralded as a positive step, this change may have created an unintended consequence for older adults with Medicare Part D insurance, which provides coverage for prescription drugs. Naloxone will no longer be covered under Part D, and enrollees are likely to experience higher out-of-pocket costs, which may create barriers to access [13]. Before the change, 600,000 Part D enrollees had received naloxone under Medicare Part D [13]. The policy change may therefore increase the risk of overdose death among older adults.

The naloxone example illustrates the need for overdose prevention research focused on older adults, who face several distinct risks with drug use [14]. Older adults have a higher burden of heart disease, which makes stimulant use more dangerous [15]. Older adults are more likely than younger persons to receive chronic pain treatment, which often involves chronic opioid use [16]. Pain is coupled with higher rates of chronic health conditions such as cancer, arthritis, and other degenerative diseases. Many of these conditions may be treated with opioids [17]. Patients aged 50 to 64 years with chronic pain have a higher risk of misusing prescription opioids compared to those without chronic pain [18]. Elderly individuals have a decreased ability to metabolize opioids with impaired renal and hepatic function, which may contribute to overdose [19]. Cognitive decline with age may lead to discrepancies in self-monitoring of drug intake, increasing the risk of overdose [20]. Older adults also have reported higher rates of polypharmacy, raising concerns for drug interactions and adverse events [21]. Furthermore, the association of depression and social isolation with substance use disorder has been well-documented and may hold greater significance for the elderly, as this population has a higher rate of depression [22]. 

Screening for substance use disorder can identify individuals at risk of overdose or harmful use. Multiple screening tools exist for a variety of substances, including alcohol, cannabis, cocaine, methamphetamines, and opioids [23, 24]. These tools are widely used, but older adults appear to be screened less often, and efficacy may be lower if screening tools are not tailored to the environments of older adults, such as social isolation and chronic health conditions [25]. On the other hand, many older adults may have more frequent contact points in the healthcare system because of the high frequency of comorbid conditions. These visits may provide “touchpoints” at which substance use education, screening, and overdose risk assessment is possible [26]. EDs serve as prominent touchpoints because older adults visit EDs at higher rates than younger persons [27]. One national study found that an average of 6.4 older adults with OUD report to the ED every hour [28]. 

Recent literature has often compared factors related to opioid overdose in younger versus older populations [29]. However, research focused within the older population is limited. One counterexample has been the description of two general patterns of substance use among the elderly: “early-onset” and “late-onset” users [30]. Early-onset users are individuals with a long history of substance use, while late-onset users are individuals who developed substance use habits at an older age. Reported causes of early-onset use include drug culture, social class, race, and drug availability during the 1960 and 1970 s. Causes of late-onset use often surround physical and mental health conditions as well as social isolation. Discovering such patterns among the older adult population can provide critical guidance for targeted interventions. Given the increasing prevalence of drug use and overdose in older adults, further investigation into the circumstances surrounding fatal overdoses is crucial. This work can better equip healthcare professionals and service providers to recognize opportunities for targeted screening, harm reduction and treatment, as well as identify older adult risk factors and pathways to improve older adult overdose prevention.

This study uses Latent class analysis (LCA) to identify distinct profiles of older adults who die by unintentional drug overdose and uses these profiles to identify potential touchpoints for prevention. LCA is a statistical method to identify profiles from a group of categorical variables [31–33]. These profiles can uncover unobserved heterogeneity in data. LCA is a powerful tool that has yet to be applied to unintentional overdose mortality in the older adult population.

## Methods

The Centers for Disease Control and Prevention’s State Unintentional Drug Overdose Reporting System (SUDORS) database for the state of Illinois provided data for this study [34]. Cases included in Illinois SUDORS are defined as those with cause of death of drug poisoning/overdose and manner of death determined as unintentional/accident and where the death occurred in Illinois. These include deaths with ICD-10 codes of Unintentional poisoning ICD-10 × 40-44 and Poisoning of undetermined intent (Y10-24 assigned. SUDORS captures 92.6% of all unintentional drug overdoses in Illinois.

SUDORS includes structured and unstructured (narrative) variables. With 1,752 unique potential variables, SUDORS provides the most detailed data on unintentional drug overdose cases in the United States. Variables include information on decedent demographics, mental and physical health problems and treatment, substance use disorder issues and treatment, family and relationship status, circumstances leading up to the overdose death, details on the overdose incident, and comprehensive toxicology. Each case includes a “narrative” that:

provides the who, what, where, when, and why of the overdose death. SUDORS data abstractors write a complete description for each overdose death detailing all components (such as cause of death, circumstances, and toxicology) in one place. These narratives provide additional context for understanding the overdose and supporting information on circumstances captured within the system. For example, if there is an indication of “previous drug overdose” in the system, the narrative might provide context about the timing of the previous overdose, drug(s) involved, and any treatment received. These narratives lend themselves to in-depth qualitative analyses of the context and circumstances of overdose deaths, which can inform prevention efforts [34].

More information on the variables included is found in the SUDORS coding manual: https://www.cdc.gov/drugoverdose/fatal/pdf/SUDORS_Coding_Manual_OD2A_v6.3.pdf.

Cases were included in this analysis if (a) the death occurred in Illinois between January 1, 2018-December 31, 2021, (b) the manner of death was unintentional drug overdose, and (c) the age of the decedent was 55 years or older. These criteria identified 2,296 decedents. We set a threshold at 55 years because adults now more commonly work into older ages, and those within 55–64 years of age have more in common with those 65 + years of age in regard to employment status [35]. Furthermore, Medicaid expansion has enabled health insurance for many adults 55–64 years of age, so their healthcare access is more comparable to those of 65 + years of age than in the past [36]. Finally, drug dependence has been shown to cause premature aging, so older adults who are biologically 55–64 years of age may present as older than their chronological years [37]. 

Decedents were analyzed across multiple variables. We selected a set of variables to identify profiles related to potential touchpoints for intervention. See Table 1 for a list of variables, definitions, and coding categories used in the analysis.

**Table 1:** List of variables used in LCA

Variable	Description	Values
sex	Biological sex of the victim	female; male
alcohol	Alcohol test result (e.g., was alcohol present)	no; yes; not tested or unknown
isolation	Coded “yes” if isolation bag of words variable or Homeless variable is coded yes	no; yes
ed	ED visit within last year	no; yes; unknown
pain_treat	Treated for acute and/or chronic pain at time of fatal overdose	no; yes; unknown
pain	Coded based on bag-of-word indicators from narrative	no; yes
race	Race category	black; white; mixed, other, or unspecified

Variables included sex, race (black, white, mixed/other), alcohol positivity, ED visit within the year preceding death, in treatment for pain at the time of their death, diagnosis of health condition associated with pain, and social isolation. Latent Class Analysis (LCA) methods were applied to identify distinct profiles of older adult decedents. LCA can identify latent classes, or distinct subgroups of decedents, based on the patterns of answers to each variable [31]. 

Independent sensitivity analyses were conducted with a reduced set of terms for the “bag of words” indicators of *isolation* and *pain*. The supplementary material contains a brief summary of all sensitivity analyses.

All analyses were conducted with R version 4.2.1, with the `depmixS4’ package for LCA. Code is available upon request [38, 39]. 

## Results

Table 2 summarizes characteristics of the 2,296 subjects in the study.

**Table 2:** Summary Statistics

Characteristic	Number	Percent
**Total**	2296	
**Sex**		
Male	1736	75.68
Female	558	24.32
**Age**		
55–64	1841	80.25
65–74	413	18.00
75–84	37	1.61
85–94	< 5	--
95–100	< 5	--
**Race**		
White	904	39.24
Black	1392	60.42
Asian	< 5	--
American Indian	5	0.22
Pacific Islander	--	--
**Drug & Alcohol involvement**		
Any opioid (yes)	1801	78.44
Fentanyl as a cause of death (yes)	1511	65.81
Only illicit opioids as a cause of death (yes)	297	12.94
Only prescription opioids a cause of death (yes)	151	6.58
Both prescription and illicit opioids as a cause of death (yes)	1330	57.92
Cocaine as a cause of death (yes)	919	40.03
Methamphetamine as a cause of death (yes)	114	4.97
Alcohol test result positive (yes)	667	33.00
**Other**		
Emergency Department Visit in last year (yes)	150	7.01
Isolation or Homeless indicators (yes)	367	15.98

*Values < 5 suppressed. *Totals may differ due to missing/unknown data

We adopted the SUDORS definition of prescription and illicit opioids as follows. “Prescription opioids include: alfentanil, buprenorphine, butorphanol, codeine, dihydrocodeine, hydrocodone, hydromorphone, levorphanol, loperamide, meperidine, methadone, morphine, nalbuphine, noscapine, oxycodone, oxymorphone, pentazocine, prescription fentanyl, propoxyphene, remifentanil, sufentanil, tapentadol, thebaine, and tramadol. Also included as prescription opioids are brand names and metabolites (e.g., nortramadol) of these drugs and their combinations with non-opioids (e.g., acetaminophen-oxycodone). Morphine is coded as prescription only if scene or witness evidence did not indicate likely heroin use and if 6-acetylmorphine was not also detected in postmortem toxicology. Fentanyl is also coded as a prescription opioid based on scene, toxicology, or witness evidence (e.g., a fentanyl patch was found at the scene). Illicit opioids include illegally-produced fentanyl, heroin, and other opioids (including synthetic opioids such as U-47700 or isotonitazene). In the absence of sufficient evidence to classify fentanyl as a prescription, fentanyl was classified as illegally made, as the vast majority of fentanyl overdose deaths involve illicit sources. All fentanyl analogs except alfentanil, remifentanil, and sufentanil (which have authorized human medical use) were included as “illegally-made fentanyl.”

Table 3 presents model fit statistics for the LCA as the number of classes grows. The log likelihood column provides the value of the log likelihood at the optimized parameters. While a single value has no interpretation on its own, the difference in the value across models can provide a measure of how fit improves with a more flexible model. The Lo-Mendell-Rubin likelihood ratio test (LMRT) uses the log likelihood to evaluate the null hypothesis that a sample is drawn from a k-class distribution against the alternative that the sample was drawn from a k + 1-class distribution [40]. The LMRT column contains the value of the statistic, while the p-value column contains its corresponding p-value.

**Table 3:** Latent class analysis model fit statistics

Model	Log likelihood	AIC	BIC	Entropy (R^2^)	LMRT	p-value
1-class	-9420.44	18862.88	18926.01	-	-	-
2-class	-9257.36	18560.72	18692.72	0.50	326.15	< 0.001
3-class	-9198.46	18466.93	18667.79	0.48	117.80	< 0.001
4-class	-9141.05	18376.11	18645.84	0.49	114.82	< 0.001

The Akaike and Bayesian information criteria (AIC and BIC, respectively) are functions of the log likelihood used to evaluate fit [41]. These functions contain a penalty term for the number of parameters. Thus, they assess the trade-off between improvements in fit against the added complexity of more general models. The minimum AIC/BIC value as a function of the number of parameters would then indicate a preferred model. Finally, entropy (R^2^) is a measure of how well latent class analysis divides observations into classes [42]. Entropy would be maximized if, for example, all observations in a three-class model had equal probabilities of membership for each class. Lower entropy therefore indicates better separation of classes. Ultimately, we selected three over four classes primarily to aid interpretability, which scholars emphasize as a central guide for parameter selection [32]. Taken as a whole, the various measures did suggest slight improvement in moving from a 3- to a 4-class model, but a 4-class model did not provide further useful distinctions in our own subjective appraisal.

**Fig. 1:**
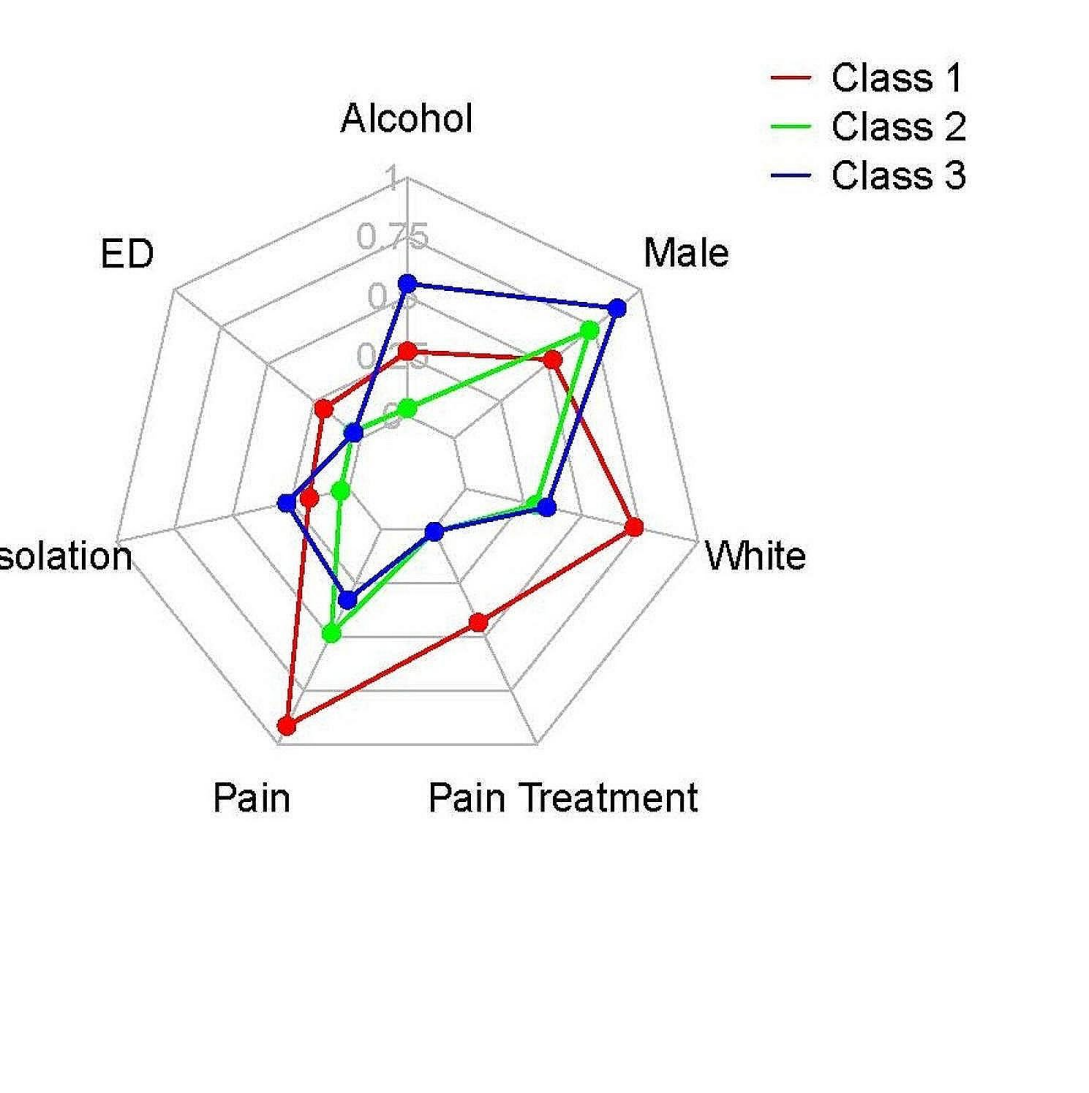
Class radar plot

Figure 1 provides a radar plot of response probabilities by class, suppressing the categories for missing values.

The numerical interpretation of Fig. 1 is contained in Table 4, which indicates the response probability or frequency of each variable conditional on each class. For example, the 0.53 response probability for `male’ in Class 1 indicates that a randomly selected member of Class 1 has a 0.53 probability of being male.

**Table 4:** Response probability for each class, as depicted in Fig. 1

Variable	Outcome	Class 1	Class 2	Class 3
male	yes	0.53	0.73	0.87
pain	yes	0.91	0.49	0.33
pain_treat	yes	0.44	0.01	0.01
	no	0.56	0.98	0.99
	missing/unknown	0.00	0.01	0.00
alcohol	yes	0.27	0.03	0.55
	no	0.68	0.73	0.42
	missing/unknown	0.05	0.24	0.03
ed	yes	0.20	0.04	0.04
	no	0.72	0.90	0.90
	missing/unknown	0.08	0.07	0.07
isolation	yes	0.17	0.04	0.27
race	white	0.73	0.30	0.35
	black	0.26	0.69	0.65
	other	0.02	0.01	0.00

Table 5 includes a written interpretation of the findings in Fig. 1.

**Table 5:** Interpretation of each latent class with frequencies of membership

Class	Description	Frequencies of membership
1	moderate on alcohol, most likely to have recent ED visit, moderate on isolation, highest on pain, most likely in pain treatment, predominantly white, most likely female	0.13
2	least likely to test positive for alcohol, moderate on recent ED visit, least isolated, moderate on pain, moderate on pain treatment, predominantly black (most), moderate on female	0.35
3	most likely positive for alcohol, least likely to have recent ED visit, most isolated, least likely to have pain, least likely to be in pain treatment, mainly Black, most likely male	0.52

Class 1, shown in red, primarily indicated a group of decedents with a greater female to male ratio, who were predominantly White, had a greater likelihood of pain treatment, pain conditions, and were more likely to have a recent ED visit. Class 2, shown in green, demonstrated mostly moderate levels of each variable, though decedents were most likely to be Black. This group also demonstrated the lowest likelihood for isolation. Class 3, shown in blue, was most likely male, most likely to be isolated, but least likely to have a pain condition or be in pain treatment. This class was also predominantly Black. The probabilities of membership shown in Table 5 assign each decedent to a class based on the single highest probability of membership. Class 1 contained 13% of the decedents, while Class 2 contained 35%, and Class 3 contained 52%. We provide an alternative summary of these relationships in supplemental materials.

## Discussion

The configuration of older adult drug overdose decedent characteristics into class profiles helps identify potential prevention and intervention touchpoints. Touchpoints are encounters that may offer an opportunity for intervention. Examples include an encounter with a health provider, social service provider, criminal justice system representative, or member of a family/friend social network [26]. Touchpoints provide an opportunity for assessment of substance use, and, if indicated, referral for treatment and harm reduction services.

Our analyses identified variation in possible touch points based on distinct class profiles. For example, decedents in Class 1 are more likely than decedents in other classes to be in pain treatment, have comorbid health conditions, and to have visited an ED in the year prior to their fatal overdose. Each of these indicators could bring individuals into healthcare encounters where screening, substance use monitoring, substance use treatment, and overdose harm reduction services may be offered and delivered. Interventions such as Screening, Brief Intervention and Referral to Treatment (SBIRT) may be offered in healthcare environments as a means to reach this population. Harm reduction services could be offered in these settings, including naloxone training and distribution, as well as the provision of fentanyl, benzodiazepine, xylazine (a powerful animal sedative) test strips and training.

Members of Class 2 were least likely to be socially isolated compared to the other classes of decedents and less likely than Class 1 members to have physical health problems, pain, or ED visits prior to their fatal overdose. Thus, older adults fitting the Class 2 profile may be less accessible through healthcare touchpoints and possibly more accessible through work and social encounters. This profile suggests potential opportunities to involve employers, coworkers, family members or social networks in prevention.

The challenges of reaching older adults who use substances through their social connections are distinct and include potential lack of awareness of signs of substance use or misuse among family and social connections, as well as stigma surrounding substance use, which can be a barrier to conversations or expressions of concern. Stigma may be particularly strong for older adults who have begun substance use again after a period of abstaining. One approach may be to incorporate education about signs of substance use risk and screening for substance use in settings where older adults gather, including social clubs, senior centers, senior assisted living settings, congregant meal services, and faith-based organizations. Screening for substance use could be provided alongside screenings for high blood pressure and diabetes at health fairs held at faith-based organizations, work events, and senior centers. The state of Florida launched the successful Project BRITE in 2004 using outreach in these contexts [43]. This approach may promote awareness and increase touchpoint encounters around opioid use among older adults in settings where older adults gather.

Class 3 appears the most difficult for which to identify potential touchpoints. This group of decedents is more likely to be male, have alcohol co-use, have no ED visits in the year prior to their fatal overdose, experience a high degree of social isolation, and have fewer comorbid health conditions. For this class, prevention opportunities may be more likely to occur through social service and outreach efforts serving persons with limited access to healthcare, and employment and social networks. Harm reduction, street outreach, and social service providers may be the venues most likely to engage this population in education, screening, treatment, and harm mitigation. However, in a study examining health service encounters of older adults who use non-medical opioids in Chicago, harm reduction providers expressed frustration in not being able to reach older adults using current street outreach methods [44]. They explained the challenge through older adults’ reluctance to associate with publicly offered substance use harm reduction services due to stigma and harm reduction services that focus on needle exchange when many older adults ingest opioids via snorting [44]. Due to these concerns, harm reduction services may best be delivered in the context of a wide range of social services, not just those limited to substance use. For example, services to navigate housing assistance, social security disability application, Medicaid/Medicare application, emergency food assistance, and other safety net social services. Harm reduction services for alcohol use may be especially relevant in this context as alcohol use was higher among this class of decedents.

The racial distributions among class membership are distinct, with White decedents primarily falling into Class 1, while Classes 2 and 3 had a larger presence of Black decedents. This result is somewhat expected because being in treatment for pain is a contributor to Class 1 membership. Black persons are both less likely to be prescribed opioids for pain and less likely to be referred for pain treatment than White persons [45]. Thus, the racial makeup of class profiles can inform equitable approaches to overdose prevention. For example, prevention via the co-prescription of opioids and naloxone may be less applicable to Classes 2 and 3 with predominately Black than members of Class 1, where White membership is more common. This approach will require consideration of class membership features through which older adults who use substances can be reached with education, screening, treatment, and harm reduction services.

## Limitation

This study is bolstered by use of detailed case-level data from SUDORS. In addition, the application of LCA to the older adult decedent group provides a unique approach to synthesizing multiple variables and identifying distinct profiles to guide interventions. Limitations include occasional missing data, as not all variables were available for all decedents. To minimize the risk of biased estimates in the LCA, we included an unknown/missing category for each incomplete variable. We also lacked information about the onset of opioid use, which would have offered further insight into patterns of decedent opioid use.

## Conclusions

The increasing rate of drug overdose death among adults 55 + is one feature of the current overdose crisis. A reversal of this trend requires targeted data to inform prevention strategies. This study provides insight into variation within the older adult overdose decedent groups and identifies three distinct classes of decedents—a novel contribution to the knowledge base. The three classes differ in important ways, such as race, involvement of alcohol, decedent sex, the presence of health conditions, pain treatment, emergency department visits, and social isolation. This information can inform the development and deployment of education, screening, treatment, and harm mitigation services for older adults.

Our study findings reinforce recommendations offered in the Treatment Improvement Protocol for Treating Substance Use Disorder in Older Adults in terms of the need to broaden the contexts in which substance use disorder screenings are offered to older adults [25]. These findings also suggest that a variety of contexts for education, screening, harm reduction, and intervention are needed to reach older adults at risk of fatal drug overdose. The variation in decedent profiles suggests education, screening, harm reduction, and interventions for older adults should take place in a variety of contexts, including health care encounters, congregate social settings, and social service delivery points. If screening is limited to health care settings, many opportunities to reach older adults who use substances are lost, as most older adults in our study, especially Black men, had limited touchpoints in healthcare settings prior to their fatal overdose.

The challenges of providing education, screening, harm reduction, and interventions to older adults through social networks are many. However, older adults may have access to more supportive social settings than adults of other ages, as there exist a plethora of organizations designed to meet the needs of older adults. These include meal delivery, senior centers, senior housing, assisted living settings, senior discount days at local retailers, and faith-based communities. Thus, there may be more opportunities to provide touchpoint intervention to older adults through these social connections. For example, older adults are more likely to participate in faith-based activities than younger adults [46]. That said, our data are limited in terms of information on connectivity to these potential touchpoints. More research is needed to assess their potential. Depending on results, prevention efforts may need to use increased connectivity to non-substance use disorder services to deliver education, screening, harm reduction, and treatment for substance use among older adults. This outreach is necessary to prevent future overdose deaths.

### Electronic supplementary material

Below is the link to the electronic supplementary material.

Supplementary Material 1

## Data Availability

The data that support the findings of this study are available from the Illinois Department of Public Health, but restrictions apply to the availability of these data, which were used under a bona fide agent agreement for the current study, and so are not publicly available. Data are however available from the authors upon reasonable request and with permission of the Illinois Department of Public Health. Please see: https://dph.illinois.gov/data-statistics/institutional-review-board.html for details regarding data requests.
