# Supplementary material for: Older adult drug overdose: an application of latent class analysis to identify prevention opportunities
Source: Harm Reduct J. 2024 Mar 13;21:61. doi: 10.1186/s12954-024-00973-4 (PMC10936079; doi:10.1186/s12954-024-00973-4)
Supplement: Supplementary file 1 — Supplementary Material 1 [file 12954_2024_973_MOESM1_ESM.docx]

Harm Reduction Journal Supplemental Materials

Table A1: Bag of words used in creation of social “isolation” and “pain” categorical variables.

| **Panel a) Isolation** | |  | |
| --- | --- | --- | --- |
| - Lived alone | - Decomposed(1) | - Separated | - Well-being check |
| - Wasn’t able to get in touch with | - Homeless | - Unknown when last seen alive | - No one had heard from |
| - Hand not heard from | - Unemployed | - Estranged |  |
| **Panel b) pain_chronic** | | | |
| - Chronic | - Neuropathy | - Replacement | - Surgery |
| - Surgery | - Arthritis | - COPD | - Suicidal Ideation |
| - Acetaminophen | - Stroke | - Motor Vehicle Accident | - Back injury |
| - Diabetes | - Back pain | - Heart disease | - Cancer |
| - Fibromyalgia | - Obesity | - Suicide attempt | - Depression |
| - Anxiety | - Cardiovascular disease |  |  |

Table A2. Probability of class membership by decedent characteristic.

| **Variable** | **Outcome** | **Class 1** | **Class 2** | **Class 3** |
| --- | --- | --- | --- | --- |
| male | yes | 0.07 | 0.31 | 0.62 |
|  | no | 0.34 | 0.47 | 0.20 |
| pain | yes | 0.26 | 0.50 | 0.25 |
|  | no | 0.01 | 0.21 | 0.77 |
| pain_treat | yes | 0.94 | 0.01 | 0.05 |
|  | no | 0.06 | 0.38 | 0.55 |
| alcohol | yes | 0.12 | 0.00 | 0.88 |
|  | no | 0.16 | 0.42 | 0.42 |
| ed | yes | 0.47 | 0.33 | 0.19 |
|  | no | 0.10 | 0.38 | 0.52 |
| isolation | yes | 0.14 | 0.00 | 0.86 |
|  | no | 0.13 | 0.42 | 0.45 |
| race | white | 0.28 | 0.27 | 0.45 |
|  | black | 0.04 | 0.40 | 0.57 |
|  | other | 0.29 | 0.59 | 0.12 |

Note: Probabilities may not sum to one due to rounding error.

Sensitivity analyses for terms included in the bag of words yielded little change in results. The first analysis removed any variation of “well-being check,” “hadn’t heard from”, and “wasn’t able to get in touch with” from the social isolation measure before running the LCA. Response probabilities and class membership rates showed negligible changes. The second analysis instead removed “cardiovascular disease”, “diabetes,” “obesity”, and “stroke” from the pain measure before running the LCA. Response probabilities again changed minimally, but the class membership rates did change somewhat. In Table 5, the frequencies of membership were 0.13, 0.35, and 0.52 for classes 1, 2, and 3, respectively. In the sensitivity analysis, those frequencies were 0.13, 0.43, and 0.45. So the removal of the four conditions potentially related to pain increased membership in class 2 by eight percentage points and reduced membership in class 3 by eight percentage points.

Lastly, the addition of opioid positivity in toxicology reports as an indicator in the LCA also left class 1 largely unchanged but further divided classes 2 and 3 based on positivity for opioids. Randomly selected members of class 2 had a probability of approximately 0.18 for testing positive, while probabilities for classes 1 and 3 had upwards of 0.9. Because most decedents did test positive for opioids, this division forced members of class 2 into class 3. Rates of respective membership across the classes were 0.13, 0.09, and 0.78. Interestingly, most of the other response probabilities were largely unchanged. However, the response probability for alcohol positivity fell by roughly 20 percentage points as members of class 2, who had relatively low rates of alcohol positivity, were pushed into class 3, which had members with much higher rates of positivity.

1. Hönigschnabl S, et al. . “Discovery of Decomposed and Mummified Corpses in the Domestic Setting--a Marker of Social Isolation?” Journal of Forensic Sciences. 2002.
